# State of the Journal of Patient-Reported Outcomes

**DOI:** 10.1186/s41687-021-00284-3

**Published:** 2021-01-20

**Authors:** Ron D. Hays, Chih-Hung Chang

**Affiliations:** 1 University of California, Los Angeles, USA; 2 Washington University School of Medicine, St. Louis, USA

The *Journal of Patient-Reported Outcomes* (JPRO) is an international, open access, multi-disciplinary journal publishing original research and review articles, brief communications, commentaries, editorials, and reviews of recent books and software advances in the field of patient-reported outcomes. The JPRO is an official journal of the International Society for Quality of Life Research (ISOQOL). The first JPRO articles were published on September 12, 2017.

Dennis Revicki and David Feeny were the founding Co-Editors-in-Chief. Dennis served until the start of 2020 and David finished at the end of 2020. They noted at the onset of the JPRO that it was “intended to complement and extend ISOQOL’s existing journal, *Quality of Life Research*” and was the “beginning of a beautiful journal” (https://jpro.springeropen.com/articles/10.1186/s41687-017-0009-2). The success of the JPRO is due to their extensive knowledge, hard work and dedication. The journal’s success also reflects the diligent contributions made by reviewers and the quality of the science in the published manuscripts.

Dennis and David are luminaries in the field of patient-reported outcomes. Both are former ISOQOL President’s Award recipients (Dennis in 2007 and David in 2010). In addition, Dennis is a former co-Editor-in-Chief of *Quality of Life Research* (2009–2017) and David served as ISOQOL president (2004–2005). We are grateful for the giant steps forward they made with the JPRO.

We hope to continue the upward trajectory of the journal and obtain the coveted impact factor soon. We encourage those doing patient-reported outcomes research to submit manuscripts and to serve as peer reviewers for the JPRO. We can only move forward with your help.

Sincerely,

Ron D. Hays and Chih-Hung Chang

JPRO Co-Editors-in-Chief

